# The Optimal Type and Dose of Exercise for Elevating Brain-Derived Neurotrophic Factor Levels in Patients With Depression: A Systematic Review With Pairwise, Network, and Dose–Response Meta-Analyses

**DOI:** 10.1155/da/5716755

**Published:** 2024-12-21

**Authors:** Zhu Yuping, Lei Tianbi, Shi Wentao, Li Yun, Zhang Guodong

**Affiliations:** ^1^Institute of Sport Science, College of Physical Education, Southwest University, Chongqing 400715, China; ^2^Department of Physical Education, Sichuan Province Science City Chunlei School, Chengdu 621054, China; ^3^College of Physical Education, Southwest University, Chongqing, China; ^4^International College, Krirk University, Bangkok 226002, Thailand

**Keywords:** BDNF, depression, dose–response network meta-analysis, exercise

## Abstract

**Background:** Reduced brain-derived neurotrophic factor (BDNF) levels have been linked to increased depression risk. While physical exercise is known to alleviate depressive symptoms and elevate BDNF levels, the effects of different exercise modalities and doses, along with their dose–response relationships, remain unclear.

**Objective:** This study aims to systematically evaluate the effects of various exercise types and doses on BDNF levels in patients with depression through pairwise meta-analysis, network meta-analysis (NMA), and dose–response NMA and to provide personalized exercise prescription recommendations.

**Methods:** A comprehensive search identified randomized controlled trials (RCTs) examining exercise's impact on BDNF levels in depression. Pairwise and NMA compared six exercise modalities: continuous aerobic exercise (CAE), resistance exercise (RE), combined aerobic and resistance exercise (AERE), yoga, Qigong, and mindfulness. Dose–response NMA was used to assess the relationships between exercise dose and BDNF levels.

**Results:** Thirty-six RCTs with 2515 participants were included. The pairwise meta-analysis indicated that all exercise interventions significantly elevated BDNF levels in patients with depression, with AERE, RE, and yoga demonstrating the most substantial effects. NMA rankings suggested that AERE was the most effective intervention, followed by RE, yoga, Qigong, mindfulness, and CAE. Dose–response NMA revealed a positive nonlinear dose–response relationship between total exercise volume and BDNF levels, with an optimal effective dose identified at ~610 METs-min/week. Beyond 1000 metabolic equivalent of tasks (METs)-min/week, increases in BDNF levels appeared to plateau. Moreover, each exercise type had distinct dose–response patterns, with RE and AERE having relatively higher optimal effective dose ranges, while CAE, yoga, Qigong, and mindfulness exhibited lower optimal ranges.

**Conclusions:** AERE, RE, and yoga are effective interventions for enhancing BDNF levels in patients with depression, with Qigong, mindfulness, and CAE being comparatively less effective. A positive nonlinear dose–response relationship between exercise volume and BDNF levels was observed. Further research is needed to refine dose–response relationships in this population.

## 1. Introduction

Depression is a prevalent mood disorder, characterized by a persistent low mood, loss of interest or pleasure, fatigue, and diminished self-esteem, among other symptoms [1]. According to the World Health Organization (WHO), depression is now one of the leading causes of disability globally, affecting more than 300 million individuals each year, which constitutes over 4% of the world population [2–4]. For affected individuals, depression not only severely compromises daily functioning and occupational productivity but may also result in social withdrawal, relationship breakdowns, and, in severe cases, suicidal ideation and behavior [5]. Depression also adversely affects the families of patients, placing substantial psychological burdens and stress on family members [6]. From a societal and economic viewpoint, the repercussions of depression are equally profound. It is estimated that depression leads to global economic losses amounting to trillions of dollars annually, largely due to reduced labor productivity, increased absenteeism, and premature retirement [7]. Consequently, the effective prevention and treatment of depression, along with efforts to alleviate its social and economic impact, have emerged as critical priorities in the fields of medicine and public health.

Brain-derived neurotrophic factor (BDNF) is a pivotal neurotrophin widely distributed across various brain regions, including the cerebral cortex, hippocampus, and hypothalamus, and is intimately involved in cognitive processes such as learning, memory, and emotional regulation [8, 9]. A substantial body of evidence suggests that individuals with depression exhibit reduced levels of BDNF, a finding consistently reported in both serum and brain tissue samples [10, 11]. This reduction in BDNF may represent a critical biological mechanism underlying the severity of depressive symptoms. A sedentary lifestyle or insufficient physical activity is strongly associated with an increased risk of developing depression [12]. Physical inactivity over extended periods has been linked to both physical and mental health deterioration. On the one hand, it contributes to conditions such as obesity, diabetes, and cardiovascular disease. On the other hand, it exacerbates mental health issues, including depression and anxiety, possibly through mechanisms involving reduced neuroplasticity and decreased levels of BDNF [13–15]. Exercise has emerged as a promising nonpharmacological intervention for depression [16]. Numerous studies have demonstrated that regular physical activity can significantly alleviate depressive symptoms and enhance the quality of life in patients with depression [17, 18]. This therapeutic effect is partly attributed to exercise-induced increases in BDNF levels [12, 19]. Both clinical trials and animal studies have shown that exercise enhances neuroplasticity and improves mood states in depressed patients by upregulating BDNF expression in various brain regions [15, 20]. However, despite the recognized benefits of exercise therapy, there are notable gaps in our understanding regarding the optimal exercise regimens for maximizing therapeutic outcomes in depression.

The comparative efficacy of different types of exercise in treating depression remains unclear [21]. Second, there is a lack of consensus on the influence of exercise dosage—including intensity, frequency, and duration—on depressive outcomes [22]. For example, studies by Jemni et al. [23] and Zarza-Rebollo et al. [24] have demonstrated that BDNF plays a critical role in mitigating depressive symptoms through exercise interventions. However, these investigations did not thoroughly explore the underlying mechanisms of BDNF or the dose–response relationship of exercise. Similarly, research by Gunnar et al. [25] and Kerling et al. [26] has shown that aerobic exercise significantly elevates BDNF levels and alleviates depressive symptoms in patients with major depressive disorder. Nevertheless, these studies were limited to a single type of exercise and did not provide a comprehensive comparison of the effects of various exercise modalities on BDNF levels and depressive symptoms. Moreover, the assessment of depression predominantly relies on self-report measures and clinical scales, which can be subjective and influenced by patients' emotional states and response biases. In contrast, BDNF serves as a biological marker, providing a more objective measure of the biological changes associated with depression [15]. Multiple meta-analyses and systematic reviews have shown that exercise interventions significantly elevate BDNF levels in patients with depression, highlighting its potential as a biomarker for the antidepressant effects of physical activity [21, 23, 24, 27]. BDNF measurement is objective and stable, with fluctuations in levels closely correlating with changes in the clinical status of depressed patients [28, 29]. This consistency underscores BDNF's utility in quantifying depression severity and assessing treatment efficacy. Despite these advancements, the current literature on the effects of various exercise types and doses on BDNF levels in depressed patients is relatively fragmented, with few systematic reviews or meta-analyses available, thereby limiting our understanding of optimal exercise strategies for depression management.

In light of these considerations, we conducted pairwise comparisons, network meta-analyses, and dose–response network meta-analyses to systematically evaluate and compare the effects of different exercise modalities and dosages on BDNF levels in patients with depression. First, pairwise meta-analyses allowed for direct comparisons between each form of exercise and control groups (CGs). Second, network meta-analyses, by synthesizing direct and indirect evidence, offered a more comprehensive assessment of the relative efficacy of various exercise interventions. Finally, dose–response network meta-analyses quantified the effects of varying exercise doses on BDNF levels, providing insights into how exercise intensity, frequency, and duration affect biological outcomes in depressed patients. These analyses led to the development of personalized exercise prescriptions tailored to different exercise modalities, offering practical recommendations for clinical application.

## 2. Methods

### 2.1. Protocol and Registration

This meta-analysis was conducted following the Preferred Reporting Items for Systematic Reviews and Meta-Analyses incorporating Network Meta-Analyses (PRISMA-NMA) guidelines and the Cochrane Handbook for Systematic Reviews of Interventions [30]. The study protocol was registered on the PROSPERO platform (Registration number: CRD42024578833).

### 2.2. Search Strategy and Study Selection

A comprehensive literature search was conducted using the PubMed, Web of Science, Scopus, and SPORTDiscus databases to identify studies evaluating the effects of various exercise modalities on BDNF levels in patients with depression. The search covered the inception of each database through August 2024, with no language restrictions applied. The search strategy primarily utilized Medical Subject Headings (MeSH) terms such as “Depression,” “Brain-Derived Neurotrophic Factor,” “Exercise,” “Mindfulness,” “Yoga,” “Qigong,” and “Tai Chi.” Detailed search strategies are provided in the Supporting Information. Three reviewers participated in the study selection process: two reviewers (Zhu Yuping and Lei Tianbi) independently screened the titles and abstracts for eligibility, and any disagreements were resolved through discussion with a third reviewer (Zhang Guodong), who has substantial expertise in this field. All references were managed and screened using EndNote X9.1 software.

### 2.3. Eligibility Criteria

The inclusion criteria for this meta-analysis were as follows:

a. Studies involving adults (age ≥ 18 years) with a clinical diagnosis of depression.
b. Comparative studies of exercise interventions, including comparisons between exercise intervention groups and CGs, as well as comparisons between different types or intensities of exercise interventions. As shown in Table 1, six different types of exercise interventions are included. To assess the effects of exercise dose across various modalities, only studies involving long-term exercise interventions (defined as a duration of 4 weeks or more) were included, excluding acute exercise intervention trials [31, 32]. Therefore, the minimum duration of the exercise intervention required for inclusion was ≥4 weeks.
c. CGs could include different types of exercise interventions or any nonexercise interventions, such as usual care or cognitive care.
d. Outcomes included changes in BDNF levels, reported in standard international units (e.g., ng/mL or pg/mL).
e. Only randomized controlled trials (RCTs) were considered.

The exclusion criteria were as follows:

a. Nonrandomized studies (including conference abstracts and experimental protocols).
b. Studies where data could not be obtained (the studies from which we were unable to obtain data will be included in the systematic review).

### 2.4. Data Extraction

The primary data extracted for this meta-analysis included the following: study authors, year of publication, sample size, gender distribution, age, body mass index (BMI), total duration of the intervention, weekly exercise frequency, duration of each exercise session, exercise dosage, changes in BDNF levels, changes in depression status, concurrent pharmacological treatments, and the country in which the study was conducted. Data extraction was performed independently by two reviewers (Zhu Yuping and Lei Tianbi), with any disagreements resolved through consultation with a third reviewer (Zhang Guodong). For studies with missing data, attempts were made to obtain the necessary information by contacting the corresponding authors. Studies for which data could not be obtained were excluded from the meta-analysis.

### 2.5. Exercise Dose Data Extraction and Calculation

The exercise dose was calculated using the formula: Exercise dose = Intensity of the specific exercise modality (metabolic equivalent of tasks [METs]) × Weekly exercise frequency × Duration of each exercise session [32]. The exercise dose was expressed in METs-min/week. The intensity of specific exercise modalities was coded according to the 2024 Adult Compendium of Physical Activities [33], a comprehensive resource that provides energy expenditure data for a wide range of daily and recreational activities. This compendium categorizes activities into domains such as household activities, occupational activities, sports and physical training, recreational activities, transportation activities, and others. Each activity is assigned a unique code number and corresponding METs values at varying intensities. Weekly exercise frequency was defined as the number of sessions participants completed per week during the exercise intervention trial. The duration of each exercise session was defined as the time spent exercising in each session. In cases where the duration of exercise sessions was progressively adjusted over the course of the intervention, the average total exercise time over the study period was used to represent the duration for each exercise session [32].

### 2.6. Risk of Bias and Certainty of Evidence

The risk of bias for all included studies was evaluated using the Cochrane Risk of Bias tool, version 2. This assessment was conducted independently by two reviewers (Zhu Yuping and Lei Tianbi), with any disagreements resolved by a third reviewer (Zhang Guodong). The overall risk of bias across studies was statistically analyzed using RevMan 5.4 software.

### 2.7. Statistical Analysis

#### 2.7.1. Pairwise Meta-Analysis and Publication Bias

For studies involving two or more exercise interventions, a pairwise meta-analysis was performed. This analysis utilized weighted averages to aggregate effect sizes, including mean differences (MDs) and standardized mean differences (SEs). Heterogeneity among studies was evaluated using the *I*^2^ statistic and the *Q*-test. Publication bias was assessed using funnel plots and Begg's or Egger's tests.

#### 2.7.2. Network Meta-Analysis (NMA)

NMA was conducted using MetaInsight v6.0.1 and R software version 4.4.1, with network plots and rank plots generated. MetaInsight, supported by R and Shiny, was utilized for frequency statistics calculations with the R package netmeta, while Bayesian statistical computations were performed using the R package gemtc. The initial NMA was carried out with MetaInsight. Subsequently, the R package “network” was employed to input relevant code and apply Bayesian Markov chain Monte Carlo (MCMC) methods to analyze and visualize results from the random-effects model. Data were assessed and processed a priori, with simulations conducted using four chains, an initial value of 2.5, a step size of 10, 25,000 iterations, and the first 5000 iterations discarded. Deviance information criterion (DIC) values were recorded. Convergence was assessed using Gelman–Rubin statistics, and surface under the cumulative ranking curve (SUCRA) values were calculated and summarized. Local inconsistency was evaluated using node-splitting methods, with DIC values compared to those from consistency models; a difference of less than 5 was considered indicative of model stability. SUCRA values were used to generate Bayesian rank plots for the six exercise interventions, comparing their therapeutic effects.

#### 2.7.3. Dose–Response NMA

A Bayesian model-based NMA (MBNMA) was conducted to assess the dose–response effects of various exercise interventions on BDNF levels. To ensure the accuracy and reliability of the predicted outcomes, BDNF levels were consistently reported as MD and SE in ng/mL. The MBNMAdose framework integrates dose–response modeling with Bayesian NMA, facilitating the modeling of diverse dose–response functions to connect potentially disparate evidence networks and improve the precision of treatment effect estimates. A random-effects model-based MBNMAdose approach was employed to evaluate the dose–response relationships of different exercise interventions. Parameters for these dose–response relationships were estimated using Bayesian methods and MCMC algorithms, such as those implemented in Just Another Gibbs Sampler (JAGS) or Stan. Various dose–response functions, including the Emax model, were utilized. Complex model fitting and diagnostics were performed using the MBNMAdose package in R, in conjunction with the rjags or rstan and coda packages, to integrate multiple data sources, capture heterogeneity, and provide dose-dependent effect estimates. Dose–response curves were subsequently visualized using the “ggplot2” package.

## 3. Results

### 3.1. Study Selection

**Figure 1:**
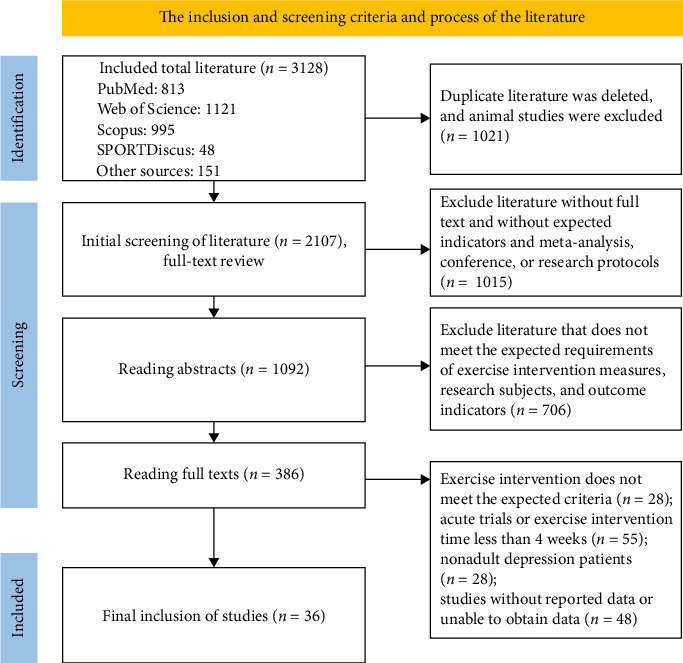
Flow diagram of the study selection.

A total of 3128 studies were initially identified from the databases based on the search strategy. After applying inclusion and exclusion criteria and removing duplicates, 214 articles remained. Full-text reviews of these 214 articles were conducted, with exclusions made for non-RCTs and studies involving interventions that did not align with the exercise types specified in Table 1. Ultimately, 36 studies were included in the analysis, all of which were previously published (Figure 1).

### 3.2. Basic Characteristics of the Included Studies

This systematic review includes six types of exercise interventions: continuous aerobic exercise (CAE), aerobic and resistance exercise (AERE), resistance exercise (RE), mindfulness, yoga, and Qigong. The specific definitions are presented in Table 1.

The demographic and study characteristics of all included studies are summarized in Table 2. These characteristics include the author, year of publication, intervention and CGs, participant age and gender, BMI, total duration of intervention (weeks), frequency of exercise sessions (sessions per week), duration of each exercise session, exercise dose (measured as METs multiplied by the total intervention period and session duration), use of antidepressant medication, and geographic region. The 36 studies included a total of 2515 participants, of whom 1551 (61.67%) were female. Six types of exercise interventions were investigated: CAE in 11 studies, AERE in six studies, RE in four studies, mindfulness exercise in five studies, yoga in five studies, and Qigong in five studies. Two studies involved both AEREs, while four studies included both mindfulness and yoga exercises. The mean duration of intervention all studies was 12.7 weeks, with an average exercise frequency of 2.7 days per week, an average session length of 54.9 min, and an average exercise dose of 627.7 METs-min/week (Table 2).

### 3.3. Risk of Bias and Publication Bias

**Figure 2:**
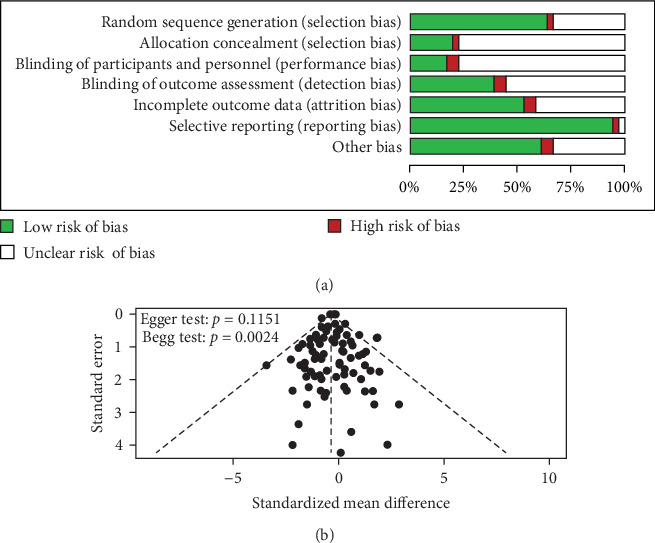
Risk assessment plots: (A) risk of bias and (B) funnel plot for publication bias. *Note:* Green indicates low risk, red indicates high risk, and blank indicates unclear risk.

The risk of bias for the 36 studies included in this meta-analysis is summarized in Figure 2. There was substantial uncertainty regarding the risks associated with allocation concealment and the blinding of participants and personnel (Figure 2A). Detailed information on bias assessment is available in the Supporting Information (Figure S1). Additionally, the funnel plot for publication bias (Figure 2B) demonstrated that while the Egger test (*p*=0.1151) did not indicate significant bias, the Begg test (*p*  < 0.05), along with the asymmetry observed in the funnel plot, suggests the presence of potential publication bias (Figure 2B).

### 3.4. Pairwise Meta-Analyses

**Figure 3:**
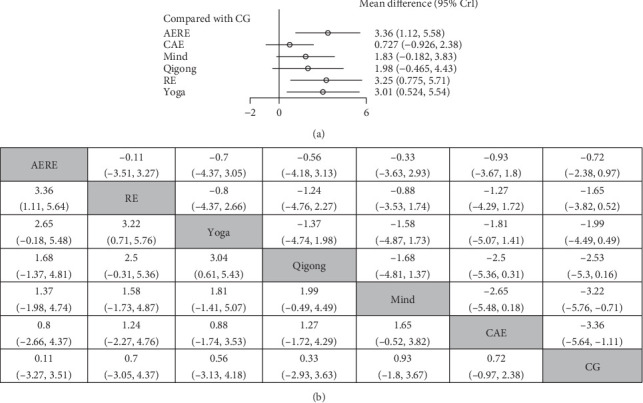
Forest plot and network ranking table of the effects of different exercise interventions on BDNF levels: (A) forest plot and (B) network ranking table. *Note:* The results of the network meta-analysis are shown in the lower left of (3B), while the results of the pairwise meta-analyses are presented in the upper right. AERE, combined aerobic and resistance exercise; BDNF, brain-derived neurotrophic factor; CAE, continuous aerobic exercise; CG, control group; RE, resistance exercise.

For patients with depression, current evidence generally suggests that a MD in BDNF levels postexercise intervention greater than 0 is associated with improvements in depressive symptoms. As shown in Figure 3A, the forest plot presents the results of pairwise comparisons between all exercise intervention groups and the CG, displayed as MDs with 95% confidence intervals (Figure 3A). Furthermore, SUCRA values were calculated using the rjags package in R, providing a ranking of intervention effects of different exercise types (Figure 3B). As illustrated in Figure 3B, compared to the CG, AERE (MD = 3.36, 95% credible interval [CrI] [1.11, 5.64]), RE (MD = 3.22, 95% CrI [0.71, 5.76]), and yoga (MD = 3.04, 95% CrI [0.61, 5.43]) were all associated with significant increases in BDNF levels among patients with depression. In addition, Qigong, mindfulness, and CAE also showed increases in BDNF levels compared to the CG, though these effects were not statistically significant (Figure 3B).

### 3.5. NMA

**Figure 4:**
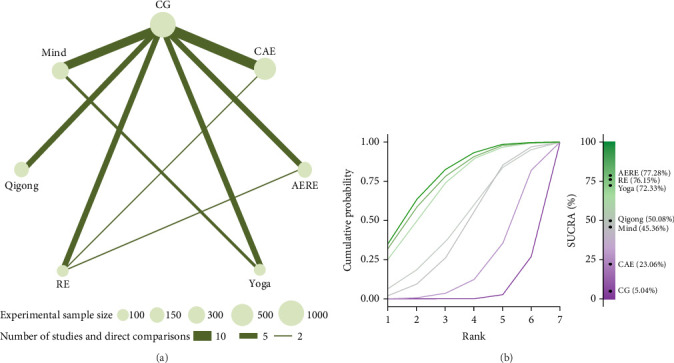
Network meta-analysis: league table and SUCRA ranking panel. (A) League table presenting the direct and indirect comparisons among different exercise interventions. (B) Bayesian ranking panel illustrating SUCRA values for each exercise intervention. (4A) displays the network of comparisons, where node sizes represent sample sizes and the thickness of the edges denotes the number of studies comparing each pair of interventions. (4B) shows the SUCRA rankings, with higher values indicating superior therapeutic effects. AERE, combined aerobic and resistance exercise; CAE, continuous aerobic exercise; CG, control group; RE, resistance exercise; SUCRA, surface under the cumulative ranking curve.

The results of the NMA are depicted in Figure 4. A comprehensive visualization of both direct and indirect comparisons among all exercise interventions and CGs is provided (Figure 4A). Additionally, a Bayesian ranking panel based on SUCRA values was constructed to evaluate the relative therapeutic efficacy of each exercise intervention (Figure 4B). Among the interventions, AERE demonstrated the highest SUCRA value (77.28%), followed by RE (76.15%), yoga (72.33%), Qigong (50.08%), mindfulness (45.36%), and CAE (23.06%) (Figure 4).

To further assess the model fit and validate the robustness of the NMA results, additional analyses were conducted. Detailed results are available in the Supporting Information. According to the Supporting Information, there was no significant difference between the random-effects model (DIC = 155.66) and the unrelated mean effects model (DIC = 148.47), based on 74 data points for BDNF (Table S1). Convergence diagnostics for all exercise interventions were satisfactory, with convergence coefficients below 1.05, indicating adequate model fit (Figure S2). Moreover, the consistency between direct and indirect evidence was evaluated using the node-splitting method, with results summarized in Table S2. No significant inconsistency was detected for most interventions (*p*  > 0.05), except for mindfulness and yoga, which exhibited a significant inconsistency (*p*=0.033 < 0.05) (Table S2). Further examination of residual deviance across all studies was performed to identify potential sources of inconsistency. The analysis, presented in Figure S4, revealed that the residual deviance for studies by Donyaei et al. [34] and Naveen et al. [35] exceeded 2. The study by Naveen et al. [35], which compared yoga with medication, may have contributed to the observed inconsistency between direct and indirect evidence for mindfulness and yoga (Figures S3 and S4). Based on these findings, a sensitivity analysis was performed, excluding studies with high residual deviance. The results and detailed descriptions of this analysis are provided in the Supporting Information (Figure S6).

### 3.6. Dose–Response NMAs

#### 3.6.1. Dose–Response NMAs of Total Exercise Volume

**Figure 5:**
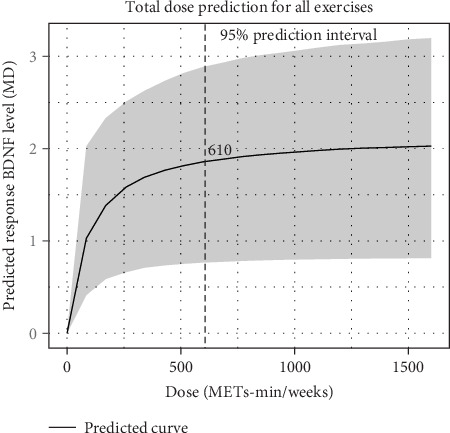
Dose–response relationship between total exercise volume and BDNF levels. The *x*-axis represents the total exercise dose, ranging from 0 to 1500 METs-min/week, while the *y*-axis shows the predicted BDNF response value at each dose level. Higher response values indicate a more pronounced effect. The solid black line illustrates the predicted response curve across different doses, depicting the trend of BDNF response as exercise volume increases. The gray shaded area represents the 95% confidence interval for the predicted response. BDNF, brain-derived neurotrophic factor; MD, mean difference; METs, metabolic equivalent of tasks.

The dose–response relationship between total exercise volume and levels of BDNF was analyzed, revealing a nonlinear association (Figure 5). As depicted in Figure 5, the predicted BDNF response increases markedly with rising exercise doses, with a significant escalation observed at ~610 METs-min/week. This finding suggests that an effective exercise dose for enhancing BDNF levels is around 610 METs-min/week. Moreover, when the exercise volume exceeds 1000 METs-min/week, the rate of BDNF level increase begins to plateau, indicating that the dose–response relationship reaches a saturation point beyond this threshold (Figure 5).

#### 3.6.2. Dose–Response NMAs of Different Exercise Interventions

**Figure 6:**
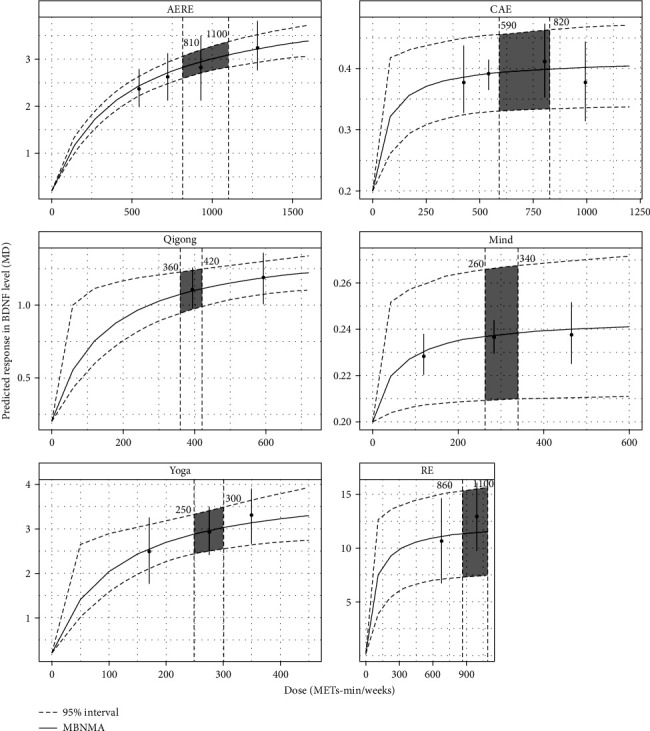
Depicts the dose–response NMA for different exercise interventions on BDNF levels. *Note:* The horizontal axis represents the dose levels of exercise interventions, while the vertical axis illustrates the predicted response of BDNF at these doses. Higher response values indicate stronger therapeutic effects. The solid black line represents the predicted response curve across various exercise doses. Dashed curves denote the 95% confidence intervals, reflecting the uncertainty in the predicted responses. Vertical curve lines show the predicted changes in the BDNF levels at specific exercise doses. The gray shaded area highlights the variability in dose effects within the key dose range. AERE, aerobic and resistance exercise; BDNF, brain-derived neurotrophic factor; CAE, continuous aerobic exercise; MBNMA, Bayesian model-based network meta-analysis; MD, mean difference; METs, metabolic equivalent of tasks; Mind, mindfulness; RE, resistance exercise.

The dose–response NMAs revealed a significant relationship between exercise dose and levels of BDNF across all six types of exercise assessed. Each intervention demonstrated a positive nonlinear dose–response relationship with BDNF levels. Among these, RE exhibited the most effective dose range, showing optimal results at 860–1100 METs-min/week. This was followed by AERE at 810–1100 METs-min/week, CAE at 590–820 METs-min/week, Qigong at 360–420 METs-min/week, mindfulness at 260–340 MET-minutes per week, and yoga at 250–300 METs-min/week (Figure 6).

To verify the robustness of the dose prediction model utilized in this study, we conducted further analyses to evaluate network connectivity among different exercise interventions and the CG. This analysis aimed to compare direct comparisons among various exercise interventions. Detailed results are provided in Figure S5 of the Supporting Information, which demonstrates extensive direct comparisons between different types of exercise interventions and the CG, thereby supporting the NMA with strong statistical evidence. Additionally, the lack of significant disconnected areas within the network suggests good connectivity, thus enhancing the accuracy and reliability of the meta-analysis. Overall, the NMA is supported by a robust dataset, facilitating reliable comparisons of the effects of different exercise interventions (Figure S5).

### 3.7. Exercise Prescription Recommendations for Patients With Depression

In light of the dose–response relationship between total physical activity and various exercise modalities' impact on BDNF levels, and guided by the 2024 Adult Compendium and WHO recommendations for physical activity, we propose preliminary exercise prescriptions for patients with depression (Table 3).

### 3.8. Sensitivity Analysis and Network Meta-Regression Analysis

To verify the reliability of the study findings and the robustness of the prediction model, we performed a sensitivity analysis on the included studies. This analysis focused on two areas: (1) excluding studies with high risk of bias and (2) excluding studies with high residuals. Detailed findings are presented in the Supporting Information (Figure S6). The sensitivity analysis demonstrated that excluding studies with high bias risk or high residuals did not alter the SUCRA rankings of the initial exercise interventions (Figure S6).

To further assess the impact of additional covariates on dose–response results, we conducted a network meta-regression analysis to examine how covariates influence dose–response NMA. Comprehensive results are detailed in the Supporting Information (Table S3). The meta-regression analysis indicates that age, BMI, intervention duration (weeks), and percentage of females do not significantly moderate the relative effects of exercise dose levels between the intervention and CGs (Table S3).

## 4. Discussion

### 4.1. Variability in the Therapeutic Effects of Different Exercise Interventions

This meta-analysis systematically evaluated the impact of six distinct exercise interventions—CAE, AERE, RE, yoga, Qigong, and mindfulness—on BDNF levels among adults with depression, assessing their relative therapeutic efficacy. Pairwise meta-analyses demonstrated that all exercise modalities significantly increased BDNF levels in patients with depression, with AERE, RE, and yoga showing particularly pronounced effects. Further ranking using NMA identified AERE as the most effective intervention, with the highest SUCRA ranking, followed by RE, yoga, Qigong, mindfulness, and CAE. It is important to note that, among the existing meta-analyses examining the relationship between exercise and BDNF levels in patients with depression, only four large-scale studies have been reported to date [21, 27, 36, 37]. These studies predominantly assessed the overall effect of exercise interventions on BDNF levels in depressed individuals, without providing comparative analyses of the therapeutic efficacy of different exercise types. Moreover, there is a notable paucity of research specifically focusing on combined exercise modalities (e.g., aerobic combined with resistance training) and low-intensity exercises (such as yoga, Qigong, and mindfulness). The present study addresses these gaps, providing a more comprehensive understanding of the differential effects of various exercise interventions on BDNF levels in this population.

The findings from both pairwise and network meta-analyses suggest that AERE significantly elevates BDNF levels in patients with depression (MD = 3.36, 95% CrI [1.11–5.64]; SUCRA, 77.28%). Previous meta-analyses have similarly demonstrated that, compared to other exercise types, a combination of aerobic and resistance training more effectively enhances BDNF levels [38, 39]. This effect may be attributed to aerobic exercise's capacity to increase systemic blood flow and oxygen delivery, thereby promoting BDNF production in the brain [40, 41]. In contrast, RE may enhance BDNF levels by increasing muscle strength, improving metabolic function, and reducing inflammatory responses [42, 43]. The synergistic effects of these two exercise modalities may thus provide more comprehensive benefits than either exercise alone. In addition to AERE, RE was identified as the second most effective intervention for increasing BDNF levels in patients with depression. The underlying mechanism may involve the ability of RE to reduce chronic inflammation and decrease the release of proinflammatory cytokines (such as TNF-*α* and IL-6), which may indirectly promote BDNF expression [42, 44]. Moreover, RE improves glucose metabolism, enhances insulin sensitivity, and regulates hormone levels (e.g., lowering cortisol), all of which may contribute to increased BDNF expression [45, 46]. Resistance training also offers the advantage of being tailored to an individual's physical condition, age, and fitness level, allowing for specific muscle group strengthening through various methods and equipment. This approach can lead to significant improvements in muscle strength and endurance in a relatively short period while effectively enhancing overall physical fitness [47, 48].

Yoga, Qigong, and mindfulness ranked next in terms of efficacy. These interventions are classified as mind–body exercises, characterized by the integration of physical, respiratory, and mental training, which together provide a holistic approach to enhancing both physical and mental well-being. Mind–body exercises can improve systemic blood circulation, enhance respiratory function, and regulate the endocrine system, thereby ensuring better nutrition and oxygen supply to the brain, which promotes neuronal health and BDNF expression [49, 50]. Additionally, these exercises improve stress management and emotional regulation skills, helping patients develop greater psychological resilience and reduce the risk of depression relapse [51, 52]. Lastly, CAE was also found to significantly improve BDNF levels in patients with depression. However, prolonged engagement in a single form of aerobic exercise may lead to increased adaptation to that exercise modality [25]. As adaptation occurs, the biochemical and physiological responses elicited by the exercise, such as BDNF release, may gradually diminish [25, 53]. This adaptation effect suggests that although aerobic exercise can initially increase BDNF levels, its efficacy may decline over time, leading to a reduced overall improvement effect. In line with recent studies, our findings show that exercise interventions have varied effects on BDNF. One study found that strength training did not significantly increase BDNF [54], contrasting with some studies in our meta-analysis where resistance training showed mixed results, possibly due to differences in population or exercise protocols. Another study demonstrated that HIIT was more effective than MICT in enhancing BDNF [55], supporting our conclusion that higher-intensity exercise may have greater neurobiological benefits for patients with depression.

### 4.2. Dose–Response Comparisons of Different Exercise Interventions

The dose–response NMAs in this study demonstrated a positive, nonlinear dose–response relationship between exercise and BDNF levels in patients with depression. To date, few meta-analyses have systematically examined the dose–response relationship between exercise dose and BDNF levels. This study is the first to establish a positive nonlinear dose–response relationship between total exercise volume and BDNF levels, identifying an optimal effective dose range for different types of exercise. Specifically, BDNF levels began to significantly increase once the total exercise volume reached 610 METs-min/week. However, beyond 1000 METs-min/week, the increase in BDNF levels plateaued, with no additional benefits observed from further increases in exercise volume. When considering the lower limit of physical activity recommended for individuals with depression by the National Health Service (NHS) guidelines (450 METs-min/week) [56] and the upper limit recommended by the WHO guidelines on physical activity and sedentary behavior (1200 METs-min/week) [57], our findings suggest that the optimal exercise dose for patients with depression may be lower than that recommended for the general population. This is consistent with current evidence indicating that patients with depression may be more sensitive to high-intensity or excessive exercise [58, 59], highlighting the need for caution when developing exercise intervention strategies.

Further examination of the dose–response relationship between various exercise interventions and BDNF levels demonstrates that all six exercise modalities (RE, AERE, CAE, Qigong, mindfulness, and yoga) elicit a positive, nonlinear effect on BDNF levels in patients with depression. However, notable variations exist in the optimal effective dose ranges across these exercise types, potentially due to several underlying factors. Firstly, different exercise modalities engage physiological systems to varying extents, thereby influencing BDNF expression. RE and AERE, for example, involve substantial muscular engagement and energy expenditure, leading to increased neuromuscular workload and metabolic stress. These conditions enhance central nervous system excitability and cerebral blood flow, thereby promoting the synthesis and release of BDNF in the brain [34, 60]. Accordingly, the optimal dose ranges for RE and AERE are relatively high (860–1100 and 810–1100 METs-min/week, respectively). Such elevated doses provide the necessary physiological stimuli to effectively elevate BDNF levels. In contrast, the optimal dose range for CAE is comparatively lower (590–820 METs-min/week). This may be related to the fact that aerobic exercise primarily improves cerebral oxygenation and metabolic function through sustained cardiovascular load and energy expenditure [25]. While aerobic exercise is effective in promoting neuroplasticity and BDNF expression, its efficacy may depend more on the cumulative duration of sustained activity rather than the intensity of individual sessions [53]. Thus, the effective dose range for aerobic exercise is somewhat lower than that for resistance training or combined modalities. Moreover, mind–body exercises such as Qigong, mindfulness, and yoga primarily achieve mental relaxation and stress reduction through techniques like meditation, deep breathing, and gentle physical movements. The dose–response curves for these interventions indicate relatively lower optimal dose ranges (Qigong: 360–420 METs-min/week; mindfulness: 260–340 METs-min/week; and yoga: 250–300 METs-min/week). Although these practices provide less direct stimulation to the musculoskeletal and cardiovascular systems, evidence suggests that they may enhance BDNF levels indirectly by modulating the autonomic nervous system and reducing the secretion of stress hormones, such as cortisol [49]. These findings imply that lower to moderate doses of these traditional exercises may effectively increase BDNF levels due to their specific impact on stress reduction and neuroplasticity. Lastly, our meta-regression analysis revealed that variables such as age, BMI, duration of intervention, and the proportion of female participants did not significantly alter the effects of exercise interventions on BDNF levels.

In summary, the observed differences in dose–response relationships may reflect the diverse physiological and psychological impacts of different exercise interventions on patients with depression. Resistance and combined training demand higher energy expenditure, which may pose challenges for patients with limited physical capacity or those new to exercise. However, these modalities are particularly effective in enhancing muscle strength and improving body composition, potentially making them more appealing to specific patient populations. Conversely, gentler exercises such as Qigong, mindfulness, and yoga, with their lower physical demands and greater emphasis on psychological relaxation, may be more suitable for patients with depression, particularly those with lower exercise capacity or motivation. These findings underscore the importance of tailoring exercise prescriptions to individual patient characteristics, disease severity, and exercise preferences in clinical practice. Future research should further investigate the long-term effects of different exercise doses and modalities and their adaptability to various subtypes of depression to optimize exercise intervention strategies.

## 5. Limitations and Future Prospects

This study adopted a comprehensive approach, utilizing pairwise meta-analysis, NMA, and dose–response meta-analysis to assess the effects of various exercise interventions on BDNF levels in patients with depression. Sensitivity analyses were conducted to exclude factors that could compromise the stability of the results, such as high risk of bias and high residuals. Additionally, network meta-regression was employed to explore the impact of potential covariates on the generalizability of the findings.

Several limitations should be acknowledged. First, there is a potential for publication bias within the data sources, and some studies lack detailed information regarding exercise dosage and patient adherence, which could influence the accuracy of the observed dose–response relationship. Second, the lack of standardization in exercise interventions and the variability in methods used to measure BDNF levels may also affect the reliability of the results. Additionally, this study did not sufficiently explore the long-term effects and potential mechanisms of action of exercise interventions, and the influence of cultural and regional differences may limit the generalizability of these findings. Future research should aim to optimize study design by increasing sample sizes and standardizing exercise interventions, employing more consistent and reliable methods for measuring BDNF levels, and further exploring the long-term effects and underlying mechanisms of exercise interventions to strengthen the evidence base.

This study focused on adult patients with depression, as adults demonstrate more stable physiological and psychological development, and the association between BDNF levels and depression is well-established, leading to more consistent and reliable findings. However, the high prevalence of depression among adolescents also demands attention. Research by Lee, Hauck, and Brooks [61] and Bjerkan et al. [62] has identified a strong relationship between physical activity and depressive symptoms in adolescents, particularly during puberty, when the incidence of depression is elevated. Future studies should further investigate the impact of exercise interventions on adolescent depression to better understand the efficacy of these interventions across age groups and to develop age-specific treatment strategies. This would provide a more targeted foundation for preventing and managing depression in adolescents.

## 6. Conclusion

This study demonstrates that a range of exercise interventions can significantly elevate BDNF levels in patients with depression, with AERE, RE, and yoga showing the most substantial effects, followed by Qigong, mindfulness, and CAE. The dose–response analysis further identifies a positive nonlinear relationship between exercise and BDNF levels and, for the first time, defines the optimal effective dose ranges for different exercise modalities. These findings lay the groundwork for developing tailored exercise prescriptions for patients with depression, offering valuable insights into optimizing exercise interventions to enhance BDNF levels in clinical settings. Nonetheless, due to the limited number of studies included in this meta-analysis, larger-scale studies are warranted to further elucidate and broaden the understanding of the relationship between exercise dosage and BDNF levels in this population.

## Figures and Tables

**Table 1:** Definitions of different exercise interventions.

Exercise	Definition
Continuous aerobic exercise	Sustained aerobic activities performed continuously over a set period, such as long-distance running or cycling

Aerobic and resistance training	A combination of aerobic exercise and resistance training within the same training plan, typically seen in comprehensive fitness programs

Resistance exercise	Training methods that utilize external resistance to induce muscle contraction, such as body weight exercises, resistance band workouts, and barbell lifting

Mindfulness	A cognitive approach and psychological state aimed at sustaining awareness of the present moment, encompassing practices such as meditation

Yoga	A holistic practice originating from ancient India, designed to achieve the harmony and unity of body and mind through various postures, breathing exercises, and meditation

Qigong	A traditional Chinese practice focused on the cultivation of the body and mind, including forms such as Tai Chi and Baduanjin

**Table 2:** Demographic and study characteristics of included studies.

Study	Interventions/control, sample size (female)	Age (mean ± SD)	BMI (mean ± SD)	Intervention duration (weeks)	Frequency	Time/session	Dose	Medication changes	Region
Zhang (2023)	CAE = 14 (12)	66.67 ± 6.04	23.64 ± 4.25	12	3	60	864	NA	China
	Walk = 14 (13)	66.22 ± 5.51	26.01 ± 3.33	12	3	60	504		
	Nonexercise = 14 (13)	69.75 ± 7.02	26.16 ± 2.73	—	3	60	0		

Reed (2022)	CAE = 44 (6)	60.0 ± 7.0	30.1 ± 6.4	12	2	40	584	Yes	Canada
	Walk = 43 (7)	61.0 ± 8.0	29.3 ± 4.9	12	2	30	168		

Cartmel (2021)	CAE = 74 (74)	57.3 ± 8.8	29.0 ± 7.2	24	4	40	1168	Yes	United States
	Attention control = 70 (70)	57.4 ± 8.5	29.1 ± 6.8	—	0	0	0		

Žlibinaitė (2020)	CAE = 13 (13)	44.2 ± 8.7	31.9 ± 3.4	24	3	50	1020	NA	Lithuania
	Nonexercise = 13 (13)	44.1 ± 5.8	32.5 ± 3.6	—	0	0	0		

Kerling (2017)	CAE = 22 (10)	44.2 ± 8.5	26.8 ± 5.1	6	3	45	675	Yes	Germany
	Usual care = 20 (6)	40.9 ± 11.9	26.8 ± 4.8	—	0	0	0		

Yeh (2015)	CAE = 41 (41)	53.2 ± 10.3	25.47 ± 1.93	12	3	50	870	Yes	China
	Nonexercise = 26 (26)	51.9 ± 11.9	24.25 ± 1.73	—	0	0	0		

Krogh (2014)	CAE = 41 (30)	38.9 ± 11.7	25.8 ± 6.4	12	3	45	810	No	Denmark
	Nonexercise = 38 (23)	43.8 ± 12.2	25.2 ± 5.1	—	0	0	0		

Schuch (2014)	CAE = 15 (11)	42.81 ± 12.4	24.78 ± 4.4	4	2	50	650	No	Brazil
	Usual care = 11 (8)	42.52 ± 13.5	25.52 ± 3.0	—	0	0	0		

Toups (2011)	CAE = 52 (52)	46.1 ± 9.5	30.3 ± 7.1	12	3	20	438	Yes	United States
	Active activities = 52 (52)	49.2 ± 9.1	31.4 ± 5.5	12	3	20	168		

Liu (2020)	CAE = 31 (5)	84.68 ± 6.74	NA	4	5	30	850	NA	China
	RE = 30 (6)	86.77 ± 6.99	NA	4	5	30	750		

Pereira (2013)	CAE = 167 (167)	70.33 ± 4.5	28.97 ± 4.79	10	3	31	976.5	Yes	Brazil
	RE = 181 (181)	71.03 ± 4.8	29.12 ± 4.80	10	3	31	725.4		

Donyaei (2024)	AERE = 19 (19)	61.3 ± 5.7	30.2 ± 1.3	12	3	60	1620	No	Iran
	Nonexercise = 18 (18)	62.1 ± 5.1	29.9 ± 1.2	—	0	0	0		

Arrieta (2020)	AERE = 57 (42)	85.1 ± 7.6	28.2 ± 5.1	24	2	60	840	NA	Spain
	Nonexercise = 55 (37)	84.7 ± 6.1	28.2 ± 5.3	—	0	0	0		

Gourgouvelis (2018)	AERE = 8 (7)	37.25 ± 8.00	28.33 ± 5.12	8	3	60	1170	Yes	Canada
	Cognitive care = 8 (5)	41.38 ± 5.66	29.25 ± 5.52	—	0	0	0		

Vedovelli (2017)	AERE = 22 (22)	83.00 ± 6.53	24.83 ± 3.76	12	3	60	1080	No	Brazil
	Cognitive care = 10 (10)	77.33 ± 9.89	24.88 ± 3.95	—	0	0	0		

Ruiz (2015)	AERE = 20 (16)	92.3 ± 2.3	25.5 ± 4.33	8	3	40	420	NA	Spain
	Nonexercise = 20 (16)	92.1 ± 2.3	27.1 ± 4.7	—	0	0	0		

Silva (2015)	AERE = 9 (0)	33.55 ± 2.63	88.24 ± 6.48	20	2	60	600	Yes	Brazil
	RE = 12 (0)	32.91 ± 2.28	83.27 ± 5.61	20	2	60	696		
	Nonexercise = 13 (0)	33.36 ± 12.19	75.56 ± 5.39	—	0	0	0		

Deus (2021)	RE = 81 (NA)	67.27 ± 3.24	27.30 ± 3.77	24	3	60	1044	NA	Brazil
	Nonexercise = 76 (NA)	66.33 ± 3.88	26.82 ± 2.90	—	0	0	0		

Church (2016)	RE high intensity = 10 (0)	23.5 ± 2.6	23.62 ± 3.73	8	4	45	1080	NA	United States
	RE low intensity = 10 (0)			8	4	45	900		

Forti (2015)	RE high intensity = 18 (10)	67.69 ± 4.3	25.63 ± 2.75	12	3	45	1012.5	NA	Belgium
	RE low intensity = 19 (10)	68.97 ± 5.1	27.92 ± 4.0	12	3	45	675		

Yarrow (2010)	RE traditional = 10 (0)	21.9 ± 0.8	25.9 ± 1.2	5	3	45	877.5	NA	United States
	RE eccentric = 10 (0)			5	3	45	675		

Liu (2024)	Mindfulness = 26 (19)	19–29	NA	8	6	45	270	No	China
	Waitlist = 30 (20)			—	0	0	0		

Guo (2022)	Mindfulness = 80 (29)	37.48 ± 11.89	NA	8	1	120	120	Yes	China
	Nonexercise = 80 (31)			—	0	0	0		

Nery (2019)	Mindfulness = 62 (62)	37.4 ± 5.3	29.8 ± 5.2	8	1	120	120	NA	Brazil
	Nonexercise = 37 (37)	37.0 ± 6.5	30.8 ± 5.7	—	0	0	0		

Carracedo (2023)	Mindfulness = 40 (NA)	18–65	NA	8	8	60	480	NA	Spain
	Tau = 10 (NA)			8	0	0	0		

Tolahunase (2018)	Mindfulness and yoga = 29 (16)	36.94 ± 8.94	26.18 ± 5.94	12	5	120	600	NA	India
	Nonexercise = 29 (15)	39.10 ± 9.26	27.10 ± 6.26	—	0	0	0		

Halappa (2018)	Yoga = 16 (7)	37.06 ± 8.08	NA	12	2	60	276	Yes	India
	Yoga and medication = 26 (10)	33.81 ± 10.77		12	2	60	276		
	Medication = 23 (10)	30.96 ± 5.94		12	0	0	0		

Naveen (2016)	Yoga = 19 (7)	35.89 ± 7.85	NA	12	2	60	276	Yes	India
	Yoga and medication = 19 (8)	34.11 ± 10.75		12	2	60	276		
	Medication = 16 (7)	33.19 ± 7.11		12	0	0	0		

Ikai (2014)	Yoga = 25 (9)	53.5 ± 9.9	24.6 ± 6.2	8	1	60	168	Yes	Japan
	Nonexercise = 25 (8)	48.2 ± 12.3	24.5 ± 3.1	—	0	0	0		

Naveen (2013)	Yoga = 19 (7)	35.9 ± 7.8	NA	12	2	60	276	Yes	India
	Yoga and medication = 22 (10)	33.6 ± 10.3		12	2	60	276		
	Medication = 21 (9)	32.4 ± 7		12	0	0	0		

Čekanauskaitė (2020)	Yoga = 18 (9)	66.9 ± 6.0	27.0 ± 4.1	10	2	90	450	NA	Lithuania
	Nonexercise = 15 (8)			—	0	0	0		

Liu b (2024)	Qigong (Baduanjin) = 50 (19)	58.86 ± 10.83	24.19 ± 3.34	8	7	60	630	NA	China
	Nonexercise = 50 (21)	56.22 ± 11.54	24.68 ± 2.86	—	0	0	0		

Li (2024)	Qigong (tai chi) = 32 (15)	62.7 ± 5.51	NA	48	2	60	396	Yes	China
	Walk = 31 (9)	61.5 ± 5.53		48	2	60	576		
	Nonexercise = 32 (13)	62.8 ± 6.14		—	0	0	0		

Sanita (2024)	Qigong = 20 (20)	62.6 ± 4.5	28.2 ± 4.4	8	3	60	594	No	China
	Nonexercise = 20 (20)	61.6 ± 7.0	25.8 ± 5.8	—	0	0	0		

Solianik (2021)	Qigong (tai chi) = 15 (13)	67.0 ± 5.9	26.0 ± 2.7	10	2	60	720	No	Lithuania
	Nonexercise = 15 (13)			—	0	0	0		

Lu (2020)	Qigong (Baduanjin) = 14 (8)	70.14 ± 7.77	NA	12	2	60	396	NA	China
	Cognitive training = 16 (6)	72.13 ± 7.16		—	0	0	0		

*Note:* In certain studies, there may be overlap in exercise interventions, such as the inclusion of mindfulness training within yoga sessions. To maintain the independence of our dose–response analysis, we excluded data from groups receiving combined interventions, retaining only those groups exposed to a single type of intervention. For transparency, the results for all groups, including those with combined interventions, are fully reported in Table 2. The BDNF levels in all included studies were measured through blood (plasma or serum) and did not use other methods (such as brain tissue extraction).

Abbreviations: AERE, combined aerobic and resistance exercise; BMI, body mass index; CAE, continuous aerobic exercise; NA, not applicable; RE, resistance exercise.

**Table 3:** Exercise prescription recommendations for patients with depression.

Exercise	Minimum effective dose (METs-min/week)^a^	Recommended energy expenditure (METs/min)^b^	Exercise frequency (sessions/week)^c^	Exercise duration (min/session)^d^
AERE	810	6.0(Code: 02145)	3–4	35–45
RE	860	6.0(Code: 02050)	3–4	35–50
CAE	590	7.3(Code: 02000)	2–3	30–40
Qigong	360	3.3(Code: 15670)	2–3	40–55
Mindfulness	260	1.0(Code: 07075)	3–4	60–80
Yoga	250	2.3(Code: 02175)	2–3	35–55

Abbreviations: AERE, combined aerobic and resistance exercise; BDNF, brain-derived neurotrophic factor; CAE, continuous aerobic exercise; METs, metabolic equivalent of tasks; RE, resistance exercise.

^a^The minimum effective dose reflects the predicted dose–response values for different exercise types on BDNF.

^b^Recommended energy expenditure is derived from the 2024 Adult Compendium, taking into account prevalent exercise interventions in the studies reviewed and selecting suitable energy expenditure plans.

^c^Exercise frequency aligns with the WHO guidelines for adult exercise, adjusted for various exercise modalities, typically two to four times per week.

^d^Exercise duration is calculated using the formula: Exercise dose = Intensity of specific exercise (METs) × Weekly exercise frequency × Duration per session. Code02145: video, exercise workouts, TV conditioning programs (e.g., cardioresistance training), vigorous; Code02050: resistance (weightlifting—free weight, nautilus, or universal type), power lifting or body building, vigorous effort (Taylor Code 210); Code02000: aerobic, general; Code15670: tai chi, qigong, general; Code07075: meditating; Code02175: yoga, general.

## Data Availability

The data that support the findings of this study are available in the paper and its supporting information of this article.

## Supporting Information

Supporting Information The supporting information includes additional results of data analysis, extended figures, detailed tables, and methods that support the findings of this manuscript. These materials are available in the Supporting Information section online.
