# Supplementary material for: The Optimal Type and Dose of Exercise for Elevating Brain-Derived Neurotrophic Factor Levels in Patients With Depression: A Systematic Review With Pairwise, Network, and Dose–Response Meta-Analyses
Source: Depress Anxiety. 2024 Dec 21;2024:5716755. doi: 10.1155/da/5716755 (PMC11919060; doi:10.1155/da/5716755)
Supplement: Supporting Information — The supporting information includes additional results of data analysis, extended figures, detailed tables, and methods that support the findings of this manuscript. These materials are available in the Supporting Information section online. [file 5716755.f1.docx]

**The Optimal Type and Dose of Exercise for Elevating Brain-Derived Neurotrophic Factor Levels in Patients with Depression: A Systematic Review with Pairwise, Network, and Dose-Response Meta-Analyses**

**Electronic Supplemental files**

**1. Search strategy**

#1 "Depression"[Mesh]

((((Depressive Symptoms[Title/Abstract]) OR (Depressive Symptom[Title/Abstract])) OR (Symptom, Depressive[Title/Abstract])) OR (Emotional Depression[Title/Abstract])) OR (Depression, Emotional[Title/Abstract])

#2 "Brain-Derived Neurotrophic Factor"[Mesh]

(((BDNF[Title/Abstract]) OR (Brain Derived Neurotrophic Factor[Title/Abstract])) OR (Factor, Brain-Derived Neurotrophic[Title/Abstract])) OR (Neurotrophic Factor, Brain-Derived[Title/Abstract])

#3 "Exercise"[Mesh]

(((((((((((((Exercise, Physical[Title/Abstract]) OR (Physical Exercise[Title/Abstract])) OR (Physical Activity[Title/Abstract])) OR (Activities, Physical[Title/Abstract])) OR (Physical Activities[Title/Abstract])) OR (Exercise, Aerobic[Title/Abstract])) OR (Aerobic Exercise[Title/Abstract])) OR (Exercises, Aerobic[Title/Abstract])) OR (Exercises, Isometric[Title/Abstract])) OR (Isometric Exercise[Title/Abstract])) OR (Acute Exercise[Title/Abstract])) OR (Exercise, Acute[Title/Abstract])) OR (Exercise Training[Title/Abstract])) OR (Training, Exercise[Title/Abstract])

#4 "Mindfulness"[Mesh]

#5 "Meditation"[Mesh]

(Transcendental Meditation[Title/Abstract]) OR (Meditation, Transcendental[Title/Abstract])

#6 "Yoga"[Mesh]

#7 "Qigong"[Mesh]

(Ch'i Kung[Title/Abstract]) OR (Qi Gong[Title/Abstract])

#8 "Tai Ji"[Mesh]

(((((((((Tai-ji[Title/Abstract]) OR (Tai Chi[Title/Abstract])) OR (Chi, Tai[Title/Abstract])) OR (Tai Chi Chuan[Title/Abstract])) OR (Taiji[Title/Abstract])) OR (Taijiquan[Title/Abstract])) OR (T'ai Chi[Title/Abstract])) OR (Tai Ji Quan[Title/Abstract])) OR (Ji Quan, Tai[Title/Abstract])) OR (Quan, Tai Ji[Title/Abstract])

#9 "Baduanjing "[Mesh]

# Risk of bias


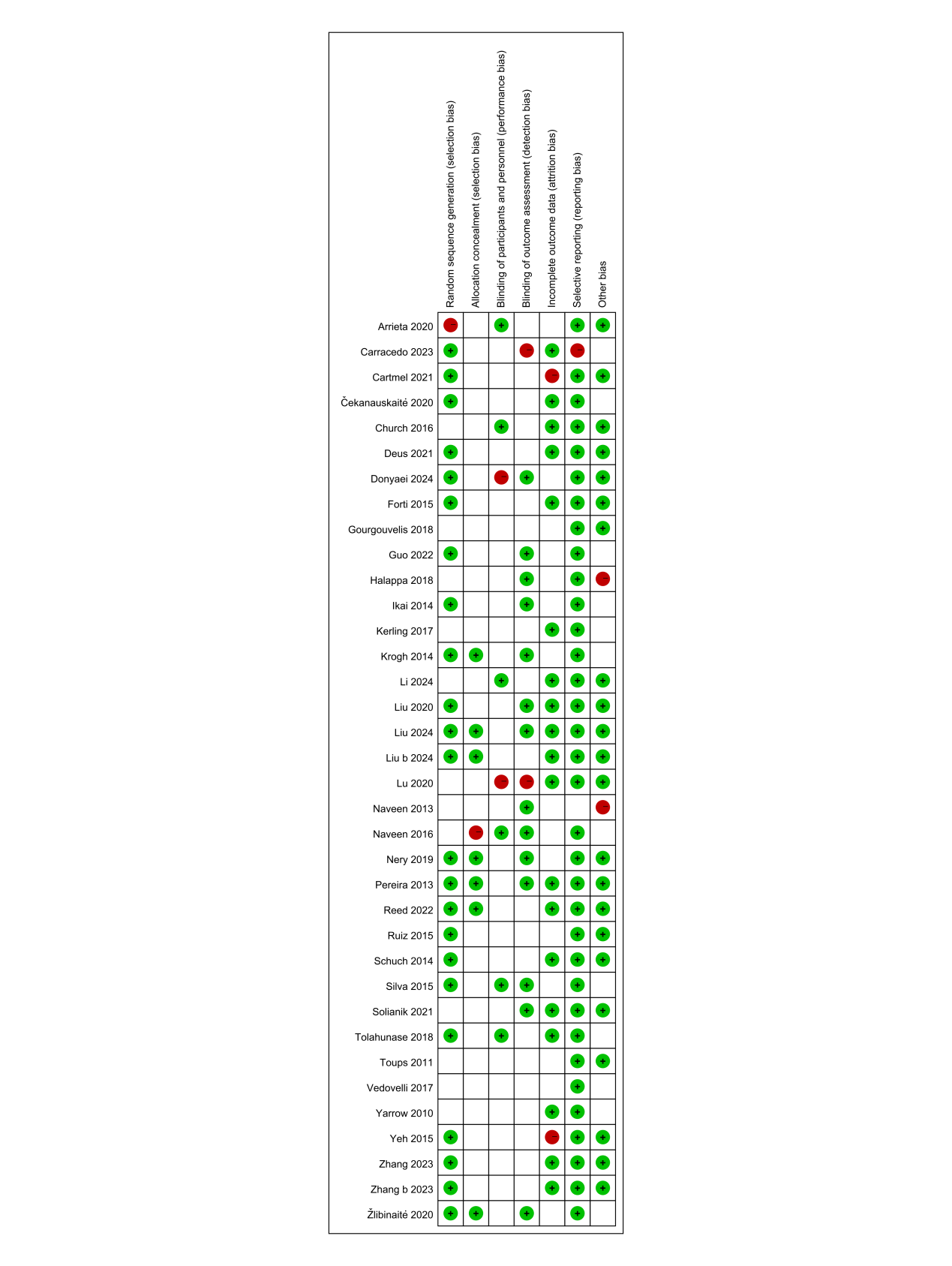


**Figure S1. Bias Risk Diagram**

Note. Each study includes six types of bias assessment, listed from left to right as follows: Random sequence generation (selection bias), Allocation concealment (selection bias), Blinding of participants and personnel (performance bias), Blinding of outcome assessment (detection bias), Incomplete outcome data (attrition bias), Selective reporting (reporting bias), and Other bias. Green indicates low risk, red indicates high risk, and blank indicates unclear risk.

**3. Network Meta-Analysis(NMA)**

## 3.1 Model fitting results

**Table S1. Model fitting results**

|  | BDNF（74 data points） | | | |
| --- | --- | --- | --- | --- |
| Model | Dbar | pD | DIC | I^2 |
| RE Model | 78.01268 | 77.65195 | 155.66462 | 6% |
| RE UME | 75.28282 | 73.19313 | 148.47595 | 3% |

Dbar, posterior mean of the deviance；DIC, deviance information criterion; pD, number of effective parameters; I^2 , Heterogeneity.

3.2 Convergence diagnosis


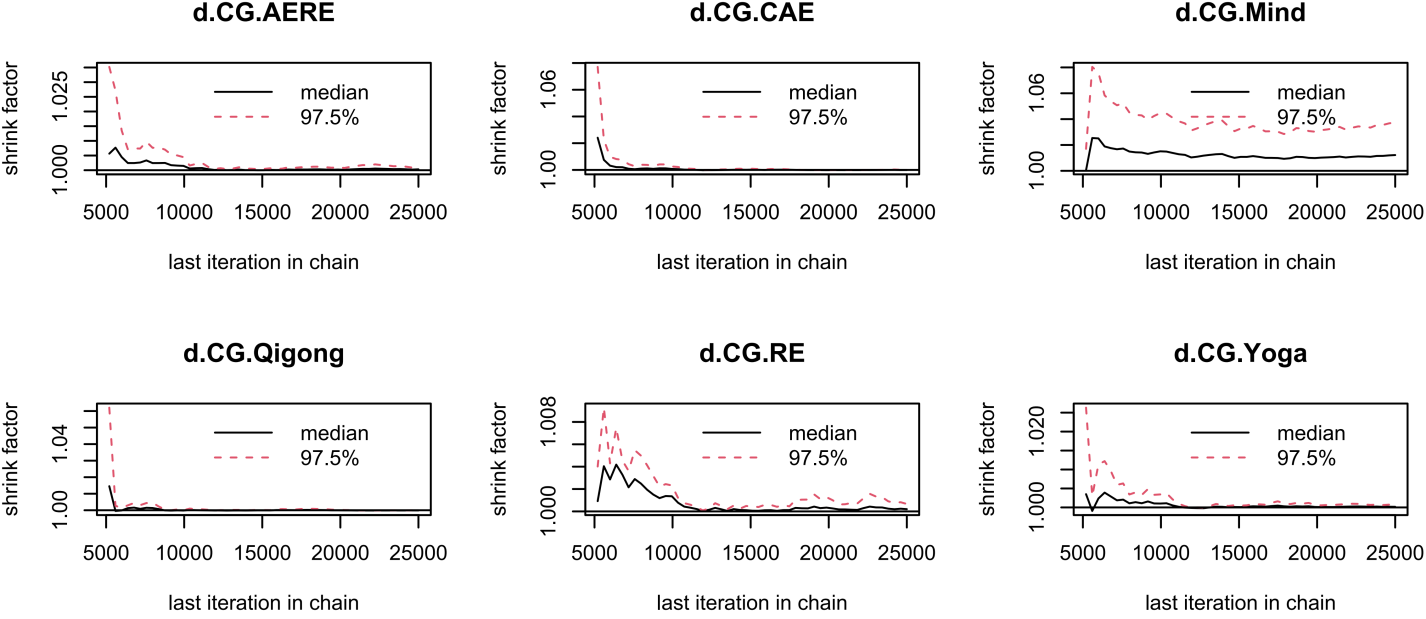


**Figure S2. The result of Convergence diagnosis**

**3.2 Node Segmentation Method**

**Table S2 Consistency Results of Direct and Indirect Evidence**

| comparison | p.value | CrI |
| --- | --- | --- |
| 1 d.AERE.RE | 0.256625 |  |
| 2 -> direct |  | -2.1 (-6.6, 2.4) |
| 3 -> indirect |  | 0.92 (-2., 3.8) |
| 4 -> network |  | 0.063 (-2.4, 2.5) |
| 5 d.CAE.CG | 0.8713 |  |
| 6 -> direct |  | 1.2 (-0.21, 2.7) |
| 7 -> indirect |  | 1.7 (-3.4, 6.7) |
| 8 -> network |  | 1.4 (-0.033, 2.8) |
| 9 d.CAE.RE | 0.88465 |  |
| 10 -> direct |  | -0.0051 (-4.5, 4.5) |
| 11 -> indirect |  | -0.36 (-2.8, 2.0) |
| 12 -> network |  | -0.26 (-2.5, 1.9) |
| 17 d.CG.RE | 0.9582 |  |
| 18 -> direct |  | -1.4 (-3.4, 0.59) |
| 19 -> indirect |  | -1.3 (-6.0, 3.4) |
| 20 -> network |  | -1.6 (-3.5, 0.24) |
| 21 d.Mind.Yoga | 0.03345 |  |
| 22 -> direct |  | 1.6 (-0.86, 4.1) |
| 23 -> indirect |  | -3.1 (-6.7, 0.45) |
| 24 -> network |  | 0.32 (-1.8, 2.5) |

**3.3 Residual results**

**3.3.1 Contribution of residual deviation of each data point**

This plot represents each data point's contribution to the residual deviance for the NMA with consistency (horizontal axis) and the unrelated mean effect (ume) inconsistency models (vertical axis) along with the line of equality. The points on the equality line means there is no improvement in model fit when using the inconsistency model, suggesting that there is no evidence of inconsistency. Points above the equality line means they have a smaller residual deviance for the consistency model indicating a better fit in the NMA consistency model and points below the equality line means they have a better fit in the ume inconsistency model. Please note that the unrelated mean effects model may not handle multi-arm trials correctly.


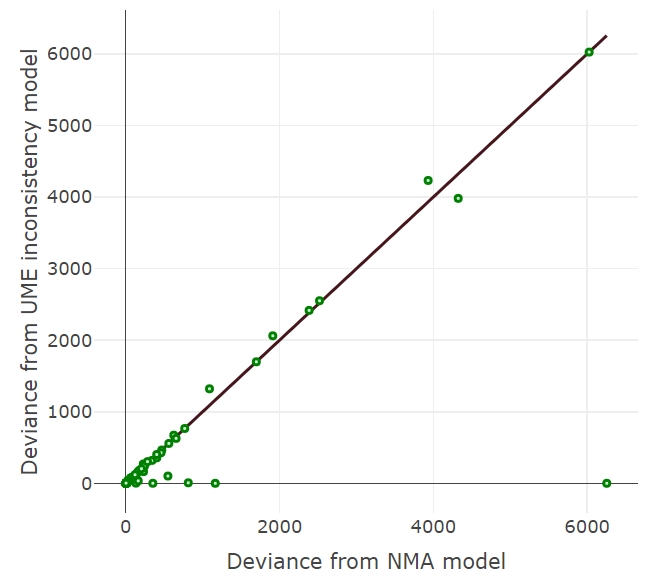


**Figure S3. Contribution of residual deviation of each data point**

**3.3.2 The posterior residual deviance per study arm**

This stem plot represents the posterior residual deviance per study arm. The total number of stems equals the total number of data points in the network meta analysis. Going from left to right, the alternating symbols on the stems indicate the different studies. Each stem corresponds to the residual deviance ($dev.ab) associated with each arm in each study. The smaller residual deviance (the shorter stem), dev.ab, the better model fit for each data point. You can identify which stem corresponds to which study arm by hovering on the stem symbols.


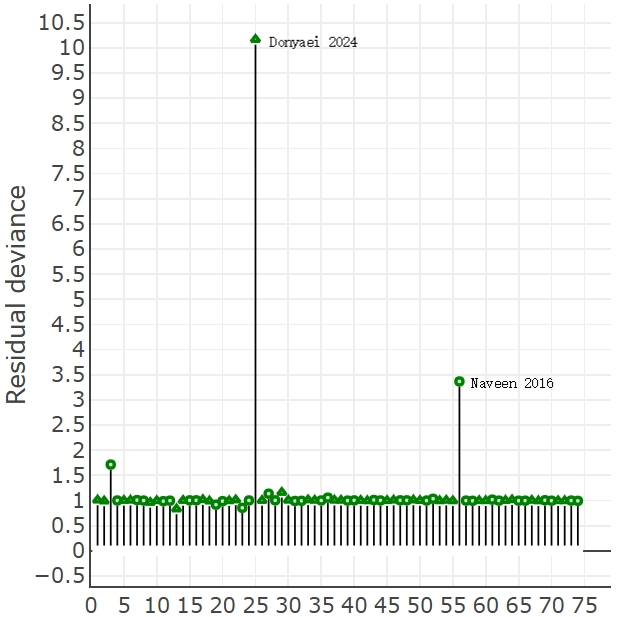


**Figure S4. residual deviation diagram of each group**

**4. Dose-Response Network Meta-Analysis**

**4.1 Network connectivity**


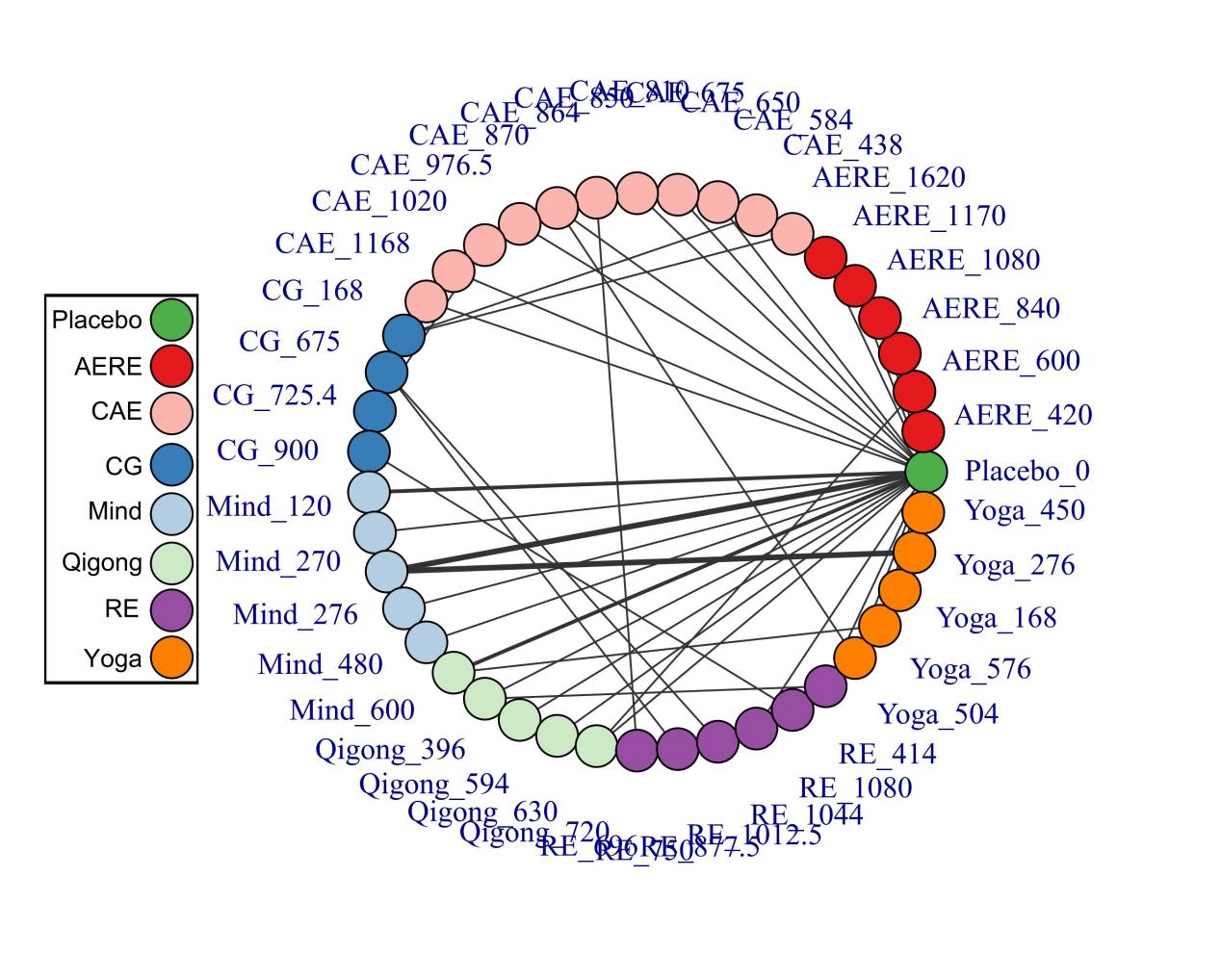


**Figure S5. network connection diagram.**

Note: Nodes (circles): Each node represents an intervention measure, including different exercise types and control groups. The color of nodes indicates different types of interventions: Node size: The size of nodes is usually related to the number of included studies or sample size. The larger the node, the higher the frequency of the intervention in the study or the larger the study sample. Connection (edge): Connection represents a direct comparison between different interventions. The thickness of the connection indicates the number of comparisons or the number of studies. The thicker the connection, the more direct comparison data there is between the two interventions. On the contrary, the thinner the connection, the less or less frequently compared data.

**5. Sensitivity analysis**

| A: Exclude high risk of bias  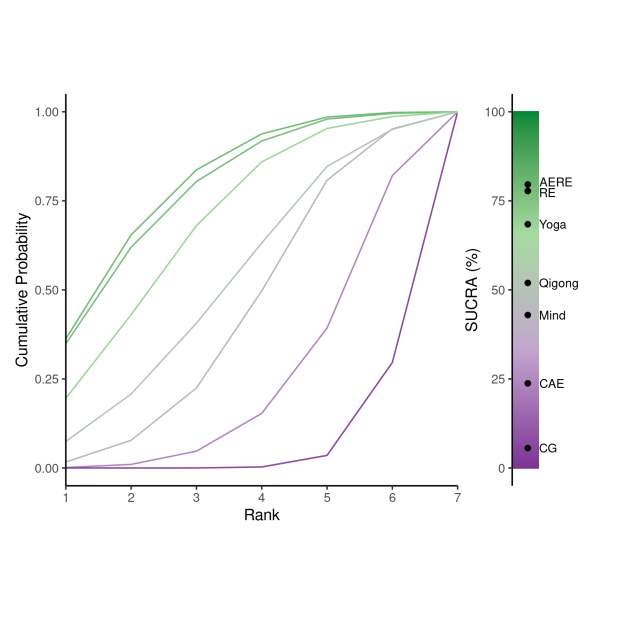 | B: Exclude high residuals  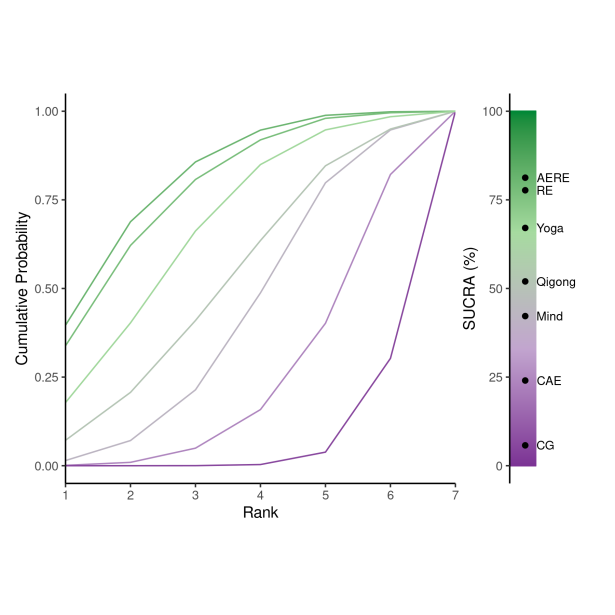 |
| --- | --- |

**Figure S6. sensitivity analysis SUCRA diagram**

**6. Network meta-regression analysis of covariates**

The effect modification for different agents versus the network reference agent can be assumed to be exchangeable/shared across the network about a common mean, ψ^

, with a between-agent standard deviation of τψ:

：ψ1,ai,k∼N(ψ^,τ2ψ)

**Table S3.** Dose response network meta-regression

| **Covariate** | **Glycosylated hemoglobin** | | | |
| --- | --- | --- | --- | --- |
|  | **DIC** | **pD** | **Deviance** | **Residual**  **Deviance** |
| Age | 285.4 | 76.2 | 216.4 | 425.8 |
| BMI | 280.1 | 69.8 | 206.2 | 416.6 |
| Intervention  duration (weeks) | 282.2 | 71.6 | 210.5 | 419.9 |
| Percent  Female | 283.6 | 73.3 | 213.3 | 422.6 |

***Note:*** BMI, body mass index. DIC, deviance information criterion; FE, fixed effects; pD, number of effective parameters; SD, standard deviation.

**7. Supplementary References for Table 2**

**Table 2. Demographic and study characteristics of included studies.**

| **Study** | **Interventions/control,**  **sample size (female)** | **Age**  **(mean±sd)** | **BMI**  **(mean±sd)** | **Intervention**  **duration**  **(weeks)** | **Frequency** | **Time**  **/session** | **Dose** | **Medication**  **changes** | **Region** |
| --- | --- | --- | --- | --- | --- | --- | --- | --- | --- |
| Zhang 2023[1] | CAE=14(12) | 66.67±6.04 | 23.64±4.25 | 12 | 3 | 60 | 864 | NA | China |
|  | Walk=14(13) | 66.22±5.51 | 26.01±3.33 | 12 | 3 | 60 | 504 |  |  |
|  | Non-exercise=14(13) | 69.75±7.02 | 26.16±2.73 | / | 3 | 60 | 0 |  |  |
| Reed 2022[2] | CAE=44(6) | 60.0±7.0 | 30.1±6.4 | 12 | 2 | 40 | 584 | YES | Canada |
|  | Walk=43(7) | 61.0±8.0 | 29.3±4.9 | 12 | 2 | 30 | 168 |  |  |
| Cartmel 2021[3] | CAE=74(74) | 57.3±8.8 | 29.0±7.2 | 24 | 4 | 40 | 1168 | YES | USA |
|  | Attention-Control=70(70) | 57.4±8.5 | 29.1±6.8 | / | 0 | 0 | 0 |  |  |
| Žlibinaitė 2020[4] | CAE=13(13) | 44.2±8.7 | 31.9±3.4 | 24 | 3 | 50 | 1020 | NA | Lithuania |
|  | Non-exercise=13(13) | 44.1±5.8 | 32.5±3.6 | / | 0 | 0 | 0 |  |  |
| Kerling 2017[5] | CAE=22(10) | 44.2±8.5 | 26.8±5.1 | 6 | 3 | 45 | 675 | YES | Germany |
|  | Usual care=20(6) | 40.9±11.9 | 26.8±4.8 | / | 0 | 0 | 0 |  |  |
| Yeh 2015[6] | CAE=41(41) | 53.2±10.3 | 25.47±1.93 | 12 | 3 | 50 | 870 | YES | China |
|  | Non-exercise=26(26) | 51.9±11.9 | 24.25±1.73 | / | 0 | 0 | 0 |  |  |
| Krogh 2014[7] | CAE=41(30) | 38.9±11.7 | 25.8±6.4 | 12 | 3 | 45 | 810 | NO | Denmark |
|  | Non-exercise=38(23) | 43.8±12.2 | 25.2±5.1 | / | 0 | 0 | 0 |  |  |
| Schuch 2014[8] | CAE=15(11) | 42.81±12.4 | 24.78±4.4 | 4 | 2 | 50 | 650 | NO | Brazil |
|  | Usual care=11(8) | 42.52±13.5 | 25.52±3.0 | / | 0 | 0 | 0 |  |  |
| Toups 2011[9] | CAE=52(52) | 46.1±9.5 | 30.3±7.1 | 12 | 3 | 20 | 438 | YES | USA |
|  | Active activities=52(52) | 49.2±9.1 | 31.4±5.5 | 12 | 3 | 20 | 168 |  |  |
| Liu 2020[10] | CAE=31(5) | 84.68±6.74 | NA | 4 | 5 | 30 | 850 | NA | China |
|  | RE=30(6) | 86.77±6.99 | NA | 4 | 5 | 30 | 750 |  |  |
| Pereira 2013[11] | CAE=167(167) | 70.33±4.5 | 28.97±4.79 | 10 | 3 | 31 | 976.5 | YES | Brazil |
|  | RE=181(181) | 71.03±4.8 | 29.12±4.80 | 10 | 3 | 31 | 725.4 |  |  |
| Donyaei 2024[12] | AERE=19(19) | 61.3±5.7 | 30.2±1.3 | 12 | 3 | 60 | 1620 | NO | Iran |
|  | Non-exercise=18(18) | 62.1±5.1 | 29.9±1.2 | / | 0 | 0 | 0 |  |  |
| Arrieta 2020[13] | AERE=57(42) | 85.1±7.6 | 28.2±5.1 | 24 | 2 | 60 | 840 | NA | Spain |
|  | Non-exercise=55(37) | 84.7±6.1 | 28.2±5.3 | / | 0 | 0 | 0 |  |  |
| Gourgouvelis 2018[14] | AERE=8(7) | 37.25±8.00 | 28.33±5.12 | 8 | 3 | 60 | 1170 | YES | Canada |
|  | Cognitive care=8(5) | 41.38±5.66 | 29.25±5.52 | / | 0 | 0 | 0 |  |  |
| Vedovelli 2017[15] | AERE=22(22) | 83.00±6.53 | 24.83±3.76 | 12 | 3 | 60 | 1080 | NO | Brazil |
|  | Cognitive care=10(10) | 77.33±9.89 | 24.88±3.95 | / | 0 | 0 | 0 |  |  |
| Ruiz 2015[16] | AERE=20(16) | 92.3±2.3 | 25.5±4.33 | 8 | 3 | 40 | 420 | NA | Spain |
|  | Non-exercise=20(16) | 92.1±2.3 | 27.1±4.7 | / | 0 | 0 | 0 |  |  |
| Silva 2015[17] | AERE=9(0) | 33.55±2.63 | 88.24±6.48 | 20 | 2 | 60 | 600 | YES | Brazil |
|  | RE=12(0) | 32.91±2.28 | 83.27±5.61 | 20 | 2 | 60 | 696 |  |  |
|  | Non-exercise=13(0) | 33.36±12.19 | 75.56±5.39 | / | 0 | 0 | 0 |  |  |
| Deus 2021[18] | RE=81(NA) | 67.27±3.24 | 27.30±3.77 | 24 | 3 | 60 | 1044 | NA | Brazil |
|  | Non-exercise=76(NA) | 66.33±3.88 | 26.82±2.90 | / | 0 | 0 | 0 |  |  |
| Church 2016[19] | RE high-intensity =10(0) | 23.5±2.6 | 23.62±3.73 | 8 | 4 | 45 | 1080 | NA | USA |
|  | RE low-intensity=10(0) |  |  | 8 | 4 | 45 | 900 |  |  |
| Forti 2015[20] | RE high-intensity=18(10) | 67.69±4.3 | 25.63±2.75 | 12 | 3 | 45 | 1012.5 | NA | Belgium |
|  | RE low-intensity=19(10) | 68.97±5.1 | 27.92±4.0 | 12 | 3 | 45 | 675 |  |  |
| Yarrow 2010[21] | RE Traditional=10(0) | 21.9±0.8 | 25.9±1.2 | 5 | 3 | 45 | 877.5 | NA | USA |
|  | RE Eccentric=10(0) |  |  | 5 | 3 | 45 | 675 |  |  |
| Liu 2024[22] | Mindfulness=26(19) | 19-29 | NA | 8 | 6 | 45 | 270 | NO | China |
|  | wait-list=30(20) |  |  | / | 0 | 0 | 0 |  |  |
| Guo 2022[23] | Mindfulness=80(29) | 37.48±11.89 | NA | 8 | 1 | 120 | 120 | YES | China |
|  | Non-exercise=80(31) |  |  | / | 0 | 0 | 0 |  |  |
| Nery 2019[24] | Mindfulness=62(62) | 37.4±5.3 | 29.8±5.2 | 8 | 1 | 120 | 120 | NA | Brazil |
|  | Non-exercise=37(37) | 37.0±6.5 | 30.8±5.7 | / | 0 | 0 | 0 |  |  |
| Carracedo 2023[25] | Mindfulness=40(NA) | 18-65 | NA | 8 | 8 | 60 | 480 | NA | Spain |
|  | TAU=10(NA) |  |  | 8 | 0 | 0 | 0 |  |  |
| Tolahunase 2018[26] | Mindfulness**＆**yoga=29(16) | 36.94±8.94 | 26.18±5.94 | 12 | 5 | 120 | 600 | NA | India |
|  | Non-exercise=29(15) | 39.10±9.26 | 27.10±6.26 | / | 0 | 0 | 0 |  |  |
| Halappa 2018[27] | Yoga=16(7) | 37.06±8.08 | NA | 12 | 2 | 60 | 276 | YES | India |
|  | Yoga**＆**medication=26(10) | 33.81±10.77 |  | 12 | 2 | 60 | 276 |  |  |
|  | medication=23(10) | 30.96±5.94 |  | 12 | 0 | 0 | 0 |  |  |
| Naveen 2016[28] | Yoga=19(7) | 35.89±7.85 | NA | 12 | 2 | 60 | 276 | YES | India |
|  | Yoga**＆**medication=19(8) | 34.11±10.75 |  | 12 | 2 | 60 | 276 |  |  |
|  | medication=16(7) | 33.19±7.11 |  | 12 | 0 | 0 | 0 |  |  |
| Ikai 2014[29] | Yoga=25(9) | 53.5±9.9 | 24.6±6.2 | 8 | 1 | 60 | 168 | YES | Japan |
|  | Non-exercise=25(8) | 48.2±12.3 | 24.5±3.1 | / | 0 | 0 | 0 |  |  |
| Naveen 2013[30] | Yoga=19(7) | 35.9±7.8 | NA | 12 | 2 | 60 | 276 | YES | India |
|  | Yoga**＆**medication=22(10) | 33.6±10.3 |  | 12 | 2 | 60 | 276 |  |  |
|  | medication=21(9) | 32.4±7 |  | 12 | 0 | 0 | 0 |  |  |
| Čekanauskaitė 2020[31] | Yoga=18(9) | 66.9 ± 6.0 | 27.0 ± 4.1 | 10 | 2 | 90 | 450 | NA | Lithuania |
|  | Non-exercise=15(8) |  |  | / | 0 | 0 | 0 |  |  |
| Liu b 2024[32] | Qigong(Baduanjin)=50(19) | 58.86±10.83 | 24.19±3.34 | 8 | 7 | 60 | 630 | NA | China |
|  | Non-exercise=50(21) | 56.22±11.54 | 24.68±2.86 | / | 0 | 0 | 0 |  |  |
| Li 2024[33] | Qigong(Tai Chi)=32(15) | 62.7±5.51 | NA | 48 | 2 | 60 | 396 | YES | China |
|  | Walk=31(9) | 61.5±5.53 |  | 48 | 2 | 60 | 576 |  |  |
|  | Non-exercise=32(13) | 62.8±6.14 |  | / | 0 | 0 | 0 |  |  |
| Sanita 2024[34] | Qigong=20(20) | 62.6±4.5 | 28.2±4.4 | 8 | 3 | 60 | 594 | NO | China |
|  | Non-exercise=20(20) | 61.6±7.0 | 25.8±5.8 | / | 0 | 0 | 0 |  |  |
| Solianik 2021[35] | Qigong(Tai Chi)=15(13) | 67.0 ± 5.9 | 26.0 ± 2.7 | 10 | 2 | 60 | 720 | NO | Lithuania |
|  | Non-exercise=15(13) |  |  | / | 0 | 0 | 0 |  |  |
| Lu 2020[36] | Qigong(Baduanjin)=14(8) | 70.14±7.77 | NA | 12 | 2 | 60 | 396 | NA | China |
|  | Cognitive training=16(6) | 72.13±7.16 |  | / | 0 | 0 | 0 |  |  |

**References:**

[1] Q. Zhang, M. Zhu, L. Huang, M. Zhu, X. Liu, P. Zhou, T. Meng, A Study on the Effect of Traditional Chinese Exercise Combined With Rhythm Training on the Intervention of Older Adults With Mild Cognitive Impairment, AM J ALZHEIMERS DIS, 38 (2023) 15333175231190626.

[2] J.L. Reed, T. Terada, L.M. Cotie, H.E. Tulloch, F.H. Leenen, M. Mistura, H. Hans, H.W. Wang, S. Vidal-Almela, R.D. Reid, A.L. Pipe, The effects of high-intensity interval training, Nordic walking and moderate-to-vigorous intensity continuous training on functional capacity, depression and quality of life in patients with coronary artery disease enrolled in cardiac rehabilitation: A randomized controlled trial (CRX study), PROG CARDIOVASC DIS, 70 (2022) 73-83.

[3] B. Cartmel, M. Hughes, E.A. Ercolano, L. Gottlieb, F. Li, Y. Zhou, M. Harrigan, J.A. Ligibel, V.E. von Gruenigen, R. Gogoi, P.E. Schwartz, H.A. Risch, L. Lu, M.L. Irwin, Randomized trial of exercise on depressive symptomatology and brain derived neurotrophic factor (BDNF) in ovarian cancer survivors: The Women's Activity and Lifestyle Study in Connecticut (WALC), GYNECOL ONCOL, 161 (2021) 587-594.

[4] L. Žlibinaitė, R. Solianik, D. Vizbaraitė, D. Mickevičienė, A. Skurvydas, The Effect of Combined Aerobic Exercise and Calorie Restriction on Mood, Cognition, and Motor Behavior in Overweight and Obese Women, J PHYS ACT HEALTH, 17 (2020) 204-210.

[5] A. Kerling, M. Kück, U. Tegtbur, L. Grams, S. Weber-Spickschen, A. Hanke, B. Stubbs, K.G. Kahl, Exercise increases serum brain-derived neurotrophic factor in patients with major depressive disorder, J AFFECT DISORDERS, 215 (2017) 152-155.

[6] S.H. Yeh, L.W. Lin, Y.K. Chuang, C.L. Liu, L.J. Tsai, F.S. Tsuei, M.T. Lee, C.Y. Hsiao, K.D. Yang, Effects of music aerobic exercise on depression and brain-derived neurotrophic factor levels in community dwelling women, BIOMED RES INT, 2015 (2015) 135893.

[7] J. Krogh, E. Rostrup, C. Thomsen, B. Elfving, P. Videbech, M. Nordentoft, The effect of exercise on hippocampal volume and neurotrophines in patients with major depression--a randomized clinical trial, J AFFECT DISORDERS, 165 (2014) 24-30.

[8] F.B. Schuch, M.P. Vasconcelos-Moreno, C. Borowsky, A.B. Zimmermann, B. Wollenhaupt-Aguiar, P. Ferrari, F.M. de Almeida, The effects of exercise on oxidative stress (TBARS) and BDNF in severely depressed inpatients, EUR ARCH PSY CLIN N, 264 (2014) 605-13.

[9] M.S. Toups, T.L. Greer, B.T. Kurian, B.D. Grannemann, T.J. Carmody, R. Huebinger, C. Rethorst, M.H. Trivedi, Effects of serum Brain Derived Neurotrophic Factor on exercise augmentation treatment of depression, J PSYCHIATR RES, 45 (2011) 1301-6.

[10] I.T. Liu, W.J. Lee, S.Y. Lin, S.T. Chang, C.L. Kao, Y.Y. Cheng, Therapeutic Effects of Exercise Training on Elderly Patients With Dementia: A Randomized Controlled Trial, ARCH PHYS MED REHAB, 101 (2020) 762-769.

[11] D.S. Pereira, B.Z. de Queiroz, A.S. Miranda, N.P. Rocha, D.C. Felício, E.C. Mateo, M. Favero, F.M. Coelho, F. Jesus-Moraleida, P.D. Gomes, A.L. Teixeira, P.L. Máximo, Effects of physical exercise on plasma levels of brain-derived neurotrophic factor and depressive symptoms in elderly women--a randomized clinical trial, ARCH PHYS MED REHAB, 94 (2013) 1443-50.

[12] A. Donyaei, E. Kiani, H. Bahrololoum, O. Moser, Effect of combined aerobic-resistance training and subsequent detraining on brain-derived neurotrophic factor (BDNF) and depression in women with type 2 diabetes mellitus: A randomized controlled trial, DIABETIC MED, 41 (2024) e15188.

[13] H. Arrieta, C. Rezola-Pardo, M. Kortajarena, G. Hervás, J. Gil, J.J. Yanguas, M. Iturburu, S.M. Gil, J. Irazusta, A. Rodriguez-Larrad, The impact of physical exercise on cognitive and affective functions and serum levels of brain-derived neurotrophic factor in nursing home residents: A randomized controlled trial, MATURITAS, 131 (2020) 72-77.

[14] J. Gourgouvelis, P. Yielder, S.T. Clarke, H. Behbahani, B.A. Murphy, Exercise Leads to Better Clinical Outcomes in Those Receiving Medication Plus Cognitive Behavioral Therapy for Major Depressive Disorder, FRONT PSYCHIATRY, 9 (2018) 37.

[15] K. Vedovelli, B.L. Giacobbo, M.S. Corrêa, A. Wieck, I. Argimon, E. Bromberg, Multimodal physical activity increases brain-derived neurotrophic factor levels and improves cognition in institutionalized older women, GEROSCIENCE, 39 (2017) 407-417.

[16] J.R. Ruiz, F. Gil-Bea, N. Bustamante-Ara, G. Rodríguez-Romo, C. Fiuza-Luces, J.A. Serra-Rexach, A. Cedazo-Minguez, A. Lucia, Resistance training does not have an effect on cognition or related serum biomarkers in nonagenarians: a randomized controlled trial, INT J SPORTS MED, 36 (2015) 54-60.

[17] B.A. Silva, R.C. Cassilhas, C. Attux, Q. Cordeiro, A.L. Gadelha, B.A. Telles, R.A. Bressan, F.N. Ferreira, P.H. Rodstein, C.S. Daltio, S. Tufik, M.T. de Mello, A 20-week program of resistance or concurrent exercise improves symptoms of schizophrenia: results of a blind, randomized controlled trial, BRAZ J PSYCHIAT, 37 (2015) 271-9.

[18] L.A. Deus, H.L. Corrêa, R. Neves, A.L. Reis, F.S. Honorato, V.L. Silva, M.K. Souza, T.B. de Araújo, A.L. de Gusmão, C.V. Sousa, T.L. Reis, L.S. de Aguiar, H.G. Simões, J. Prestes, G.F. Melo, T.S. Rosa, Are Resistance Training-Induced BDNF in Hemodialysis Patients Associated with Depressive Symptoms, Quality of Life, Antioxidant Capacity, and Muscle Strength? An Insight for the Muscle-Brain-Renal Axis, INT J ENV RES PUB HE, 18 (2021).

[19] D.D. Church, J.R. Hoffman, G.T. Mangine, A.R. Jajtner, J.R. Townsend, K.S. Beyer, R. Wang, M.B. La Monica, D.H. Fukuda, J.R. Stout, Comparison of high-intensity vs. high-volume resistance training on the BDNF response to exercise, J APPL PHYSIOL, 121 (2016) 123-8.

[20] L.N. Forti, E. Van Roie, R. Njemini, W. Coudyzer, I. Beyer, C. Delecluse, I. Bautmans, Dose-and gender-specific effects of resistance training on circulating levels of brain derived neurotrophic factor (BDNF) in community-dwelling older adults, EXP GERONTOL, 70 (2015) 144-149.

[21] J.F. Yarrow, L.J. White, S.C. McCoy, S.E. Borst, Training augments resistance exercise induced elevation of circulating brain derived neurotrophic factor (BDNF), NEUROSCI LETT, 479 (2010) 161-5.

[22] W. Liu, J. Yuan, Y. Wu, L. Xu, X. Wang, J. Meng, Y. Wei, Y. Zhang, C.Y. Kang, J.Z. Yang, A randomized controlled trial of mindfulness-based cognitive therapy for major depressive disorder in undergraduate students: Dose- response effect, inflammatory markers and BDNF, PSYCHIAT RES, 331 (2024) 115671.

[23] H. Guo, Y. Ren, B. Huang, J. Wang, X. Yang, Y. Wang, Psychological Status, Compliance, Serum Brain-Derived Neurotrophic Factor, and Nerve Growth Factor Levels of Patients with Depression after Augmented Mindfulness-Based Cognitive Therapy, GENET RES, 2022 (2022) 1097982.

[24] S.F. Nery, S. Paiva, É.L. Vieira, A.B. Barbosa, E.M. Sant'Anna, M. Casalechi, C.C. Dela, A.L. Teixeira, F.M. Reis, Mindfulness-based program for stress reduction in infertile women: Randomized controlled trial, STRESS HEALTH, 35 (2019) 49-58.

[25] D. Carracedo-Sanchidrian, C. de Dios-Perrino, C. Bayon-Perez, B. Rodriguez-Vega, M.F. Bravo-Ortiz, M.Á. Ortega, A.M. González-Pinto, G. Lahera, Effect of mindfulness-based cognitive therapy vs. psychoeducational intervention on plasma brain-derived neurotrophic factor and cognitive function in bipolar patients: a randomized controlled trial, FRONT PSYCHIATRY, 14 (2023) 1279342.

[26] M.R. Tolahunase, R. Sagar, M. Faiq, R. Dada, Yoga- and meditation-based lifestyle intervention increases neuroplasticity and reduces severity of major depressive disorder: A randomized controlled trial, Restor Neurol Neurosci, 36 (2018) 423-442.

[27] N.G. Halappa, J. Thirthalli, S. Varambally, M. Rao, R. Christopher, G.B. Nanjundaiah, Improvement in neurocognitive functions and serum brain-derived neurotrophic factor levels in patients with depression treated with antidepressants and yoga, INDIAN J PSYCHIAT, 60 (2018) 32-37.

[28] G.H. Naveen, S. Varambally, J. Thirthalli, M. Rao, R. Christopher, B.N. Gangadhar, Serum cortisol and BDNF in patients with major depression-effect of yoga, INT REV PSYCHIATR, 28 (2016) 273-8.

[29] S. Ikai, T. Suzuki, H. Uchida, J. Saruta, K. Tsukinoki, Y. Fujii, M. Mimura, Effects of weekly one-hour Hatha yoga therapy on resilience and stress levels in patients with schizophrenia-spectrum disorders: an eight-week randomized controlled trial, J ALTERN COMPLEM MED, 20 (2014) 823-30.

[30] G.H. Naveen, J. Thirthalli, M.G. Rao, S. Varambally, R. Christopher, B.N. Gangadhar, Positive therapeutic and neurotropic effects of yoga in depression: A comparative study, INDIAN J PSYCHIAT, 55 (2013) S400-4.

[31] A. Čekanauskaitė, A. Skurvydas, L. Žlibinaitė, D. Mickevičienė, S. Kilikevičienė, R. Solianik, A 10-week yoga practice has no effect on cognition, but improves balance and motor learning by attenuating brain-derived neurotrophic factor levels in older adults, EXP GERONTOL, 138 (2020) 110998.

[32] Y. Liu, C. Chen, Du H, M. Xue, N. Zhu, Impact of Baduanjin exercise combined with rational emotive behavior therapy on sleep and mood in patients with poststroke depression: A randomized controlled trial, MEDICINE, 103 (2024) e38180.

[33] G. Li, P. Huang, S. Cui, Y. He, Q. Jiang, B. Li, Y. Li, J. Xu, Z. Wang, Y. Tan, S. Chen, Tai Chi improves non-motor symptoms of Parkinson's disease: One-year randomized controlled study with the investigation of mechanisms, PARKINSONISM RELAT D, 120 (2024) 105978.

[34] S. Singsanan, N. Luangpon, S. Kiatkulanusorn, P. Boonsiri, M. Burtscher, K. Klarod, Qigong Training Effects on Brain-Derived Neurotrophic Factor and Cognitive Functions in Sedentary Middle-Aged and Elderly Females With Type 2 Diabetes, Women in Sport and Physical Activity Journal, 32 (2024) wspaj.2024-0041.

[35] R. Solianik, D. Mickevičienė, L. Žlibinaitė, A. Čekanauskaitė, Tai chi improves psychoemotional state, cognition, and motor learning in older adults during the COVID-19 pandemic, EXP GERONTOL, 150 (2021) 111363.

[36] E.Y. Lu, P. Lee, S. Cai, W. So, B. Ng, M.P. Jensen, W.M. Cheung, H. Tsang, Qigong for the treatment of depressive symptoms: Preliminary evidence of neurobiological mechanisms, INT J GERIATR PSYCH, 35 (2020) 1393-1401.
